# Examining the Importance of Hand Hygiene Policy and Patient Safety Culture on Improving Healthcare Workers’ Adherence to Hand Hygiene Practice in Critical Care Settings in the Sultanate of Oman: A Scoping Review

**DOI:** 10.7759/cureus.19773

**Published:** 2021-11-20

**Authors:** Khalid M Al Sawafi

**Affiliations:** 1 Hospital Administration, Ibra Hospital, Ibra, OMN

**Keywords:** health care quality assurance, world health organization, nosocomial infection, healthcare-associated infections, hand hygiene

## Abstract

Several studies suggest that adherence to hand hygiene (HH) policy would be enhanced by improving the culture of safety in an organization. This could be achieved through continuous awareness programs about the dramatic effect of HH practice according to the HH policy on improving patient safety and quality care. Understanding the importance and purposes of HH policy by healthcare workers would allow them to prioritize HH policy in their planning. Therefore, healthcare leaders should be responsible and accountable for strengthening their healthcare system by improving infrastructure, providing adequate support and resources, providing comprehensive monitoring and evaluation of patient safety initiatives, monitoring adherence to the regional Gulf Cooperation Council (GCC) and local Oman HH policy and using World Health Organization (WHO) guidelines for patient safety and HH as a basis for providing safer care. This should involve HH policy as a basic and mandatory program during an internship or in new staff orientation programs, spending enough resources on conducting more research studies and benchmarking findings with other international countries or any other organization such as WHO or Centres for Disease Control (CDC). The development of an HH policy at three different levels, macro, meso, and micro, is discussed in this article. In this sense, patient safety and quality care are the most important issues when adopting any policy.

## Introduction and background

Patient safety is an important matter in global health care settings. Through research studies, it was estimated that 10% of all inpatient admissions were harmed by healthcare providers, without intention, and 75% were preventable. Additionally, unsafe health care practices resulted in a heavy economic health care cost, and 5% to 10% of health care expenditure is due to unsafe practices [1]. The Institute of Medicine reported that patient safety incidents are mainly caused by human error incidents [2]. An error is defined as a ‘‘failure to carry out a planned action as intended or application of an incorrect plan’’ [3]. However, the International Classification for Patient Safety, which was initiated by WHO, defined an incident as ‘‘an event or circumstance which could have resulted or done result in unnecessary harm to patients’’ [4]. Thus, the culture of patient safety has been recognized by WHO as a concern that has been prioritized in WHO safety programs [5]. The culture of safety was defined by the European Society for Quality in Health Care in 2006 as: ‘‘An integrated pattern of individual and organizational behavior, based upon shared beliefs and values that continuously seeks to minimize patient harm which may result from the process of care delivery’’ [6].

WHO has launched many programs to implement patient safety standards, for instance, patient-friendly hospital initiatives programs and patient safety curriculum guides [7-8]. Nosocomial infection is defined by WHO as "infections acquired during hospital care which are not present or incubating at admission" [9]. Protecting patients from nosocomial infection is every health care provider's responsibility and in Oman, the Ministry of Health (MoH) has pointed out, in the Code of Professional Conduct for nurses and midwives (element number 7), the significance of risk reduction in patient safety [10]. However, as the risk of infection transmission increases with person-to-person contact during nursing and doctor's care generally and in critical care settings, such as Intensive Care Unit (ICU) and Emergency Room (ER), hand hygiene (HH) becomes essential [11]. Nevertheless, staff adherence to hand hygiene is still below the average at 38.7% according to the WHO [12]. Therefore, hands must be decontaminated through hand hygiene, and every health care provider should adhere to hand hygiene practice [13]. Although there are a variety of reasons that make staff noncompliant with hand hygiene, as identified in Table 1, following its policy must be achieved and standardized.

**Table 1:** Factors leading to healthcare providers noncompliance with hand hygiene [11]

Lack of appropriate accessible equipment
High nurse-to-patient ratio
Allergies to hand washing products
Insufficient knowledge of the nurse about the risks and procedures
Too long a duration recommended for washing and the time required

The aim of this review is therefore to examine the impact of hand hygiene policy and patient safety culture on improving healthcare workers’ adherence to hand hygiene practice in critical care settings. Thus, the current Oman hand hygiene policy and its actual impact on healthcare quality are to be critically analyzed using current evidence.

## Review

Methodology

Systematic reviews and randomized control trials (RCT) are considered to be at the top of the hierarchy of evidence according to Holland and Rees (2010). Additionally, qualitative studies, policy documents, and healthcare reports were also used although the evidence is weaker [14]. Although the search included a timescale of 10 years (starting from the year 2008), some evidence studies were older because they are considered to be 'seminal' or 'classical' studies. The keywords used for database search were: hand hygiene, healthcare-associated infections, nosocomial infection, World Health Organization, and Health Care Quality Assurance. The databases used for literature review were: Cumulative Index to Nursing & Allied Health Literature (CINAHL), Medline, PubMed, Scopus, Google Scholar, and Science Direct.

Examining the impact of HH policy in improving healthcare workers’ adherence to its practice is a global issue because HH is a central aspect in preventing healthcare-associated infections or nosocomial infections. Moreover, adherence to HH practice (with water and soap or by alcohol-based hand rub gel) is an essential factor in reducing healthcare expenditure worldwide [15-16]. Historically, the concept of HH came out in the nineteenth century. Chloride solution was used in 1822 by a French pharmacist Antoine-Germain Labarraque [17]. Then, in 1846, Ignaz Semmelweis (Hungarian physician) who discovered through his seminal study that physicians and their students were transmitting infection between mothers through their hands, leading to higher maternal mortality rate, and insisted that they should clean their hands with a chlorinated lime solution between each [18]. As a result, the number of maternal deaths dramatically reduced. For that reason, Semmelweis's observation and intervention indicated and evidenced the importance of hand decontamination with an antiseptic agent between patients because it decreases healthcare-associated infections and reduces mortality and morbidity rates. Years later, HH training programs were started in countries like the UK and the USA; in the USA, for instance, hand-washing techniques training began in public health care services In 1961. HH written guidelines were issued by the Centre for Disease Prevention and Control (CDC) in 1975, which were then published in 1981 [19]. In 1988 and 1995 the CDC HH guidelines were revised and updated by the Association for Professionals in Infection Control and Applied Epidemiology (APIC), and in 2002 alcohol-based hand rub was made [20].

However, standardizing HH practices through policy development was recognized as a measure to reduce infection and was adopted by many countries globally [12]. In Oman, HH policy has been introduced through different stages as follows: macro-level (the global and international level; i.e. WHO HH policy), meso or 'middle' level which is at the regional and national level; i.e. Gulf Cooperation Council (GCC) HH policy, and micro-level (at the hospital level; i.e. Hospital HH policy) [21]. It could be stated, however, that HH started from the 'micro' level when Semmelweis recommended through his classical study the use of an antiseptic agent between handling each patient in 1846 [22]. Conversely, in Oman, the HH policy has moved in a different direction as mentioned above, from macro to meso to micro-level (GCC 2009). The policy is not something that is particularly distinguished as a specific phenomenon, nor is it a solid one [23]. The policy could be seen as "a course of action or decision network". Hence, HH policy could be defined as a set of actions that could be performed using water and soap or by alcohol-based hand rub gel [16]. Likewise, HH policy should be enclosed with a set of processes that have resulted from problems encountered over some time. It is for this reason that analyzing HH policy would clarify its positive advantages on healthcare quality improvement.

The next section will critically analyze and synthesize the Oman HH policy using evidence from related articles to achieve the study’s aim; thus, three main themes will be used to discuss the policy: macro level, 'meso' or middle level, and micro level. However, to enrich the study's aim about HH policy, attention will be drawn to the essence of macro and meso levels in improving HH practices among healthcare workers in general, and the micro-level will focus on the impact of HH policy on staff adherence to HH in critical care settings.

Macro Level

In this article, the macro-level consideration is focused on the global and international HH policy. WHO, for example, has produced universal HH guidelines based on different studies and outcomes [12]. As such, the Oman HH policy, which initially was the same policy in the other Gulf Cooperation Council (GCC) countries (Qatar, KSA, UAE, Bahrain, and Kuwait), has referenced the WHO HH guidelines and the Association for Professionals in Infection Control and Epidemiology (APIC), both of which are internationally recognized organizations [21]. The WHO promoted several HH programs and campaigns, in 2005, for instance, the "My five moments for hand hygiene" campaign started and different HH performances from different healthcare settings were applied [16].

Moreover, in October 2005, WHO launched the first global safety challenge program under the theme 'Clean Care is Safer Care' [24]. The goal of this campaign, according to WHO (2009b), was to have universal appreciation about infection control (IC) generally and HH specifically as an essential basis of patient safety, and to promote better HH practices among healthcare workers. Thus, the policy aimed to improve compliance to HH among healthcare workers with the effect that the rates of healthcare-associated infections would reduce. The WHO has supported its HH guidelines by evidence studies. One of these studies focused on the developing countries such as Oman, as a WHO priority on IC broadly and HH explicitly [25]. The main goal was to promote patient safety in the developing countries through adherence to 'clean care'; however, the main patient safety challenges in developing countries by various problems were characterized by WHO as identified in Table 2. Consequently, these challenges (see Table 2) have created a gap between patient safety and prevention strategies; one of these strategies is HH policy, hence, strengthening the infrastructure of the healthcare system in developing countries through continuous support is essential and would result in better patient safety.

**Table 2:** Characteristics of the main patient safety challenges Source: [26]

Serial Number	Characteristics of Patient Safety Challenges
01	It affects a very large number of patients worldwide each year and has a potential impact on patients, families, and healthcare systems.
02	Methods exist to assess the size of the nature of the problem and thus create a basis for monitoring of action.
03	Infection is frequently due to multiple causes related to the system and the processes of care provision, economic constraints on systems and countries, and human behavior.
04	Solutions and interventions to prevent healthcare-associated infection exist and most are simple and inexpensive.
05	Several hospitals and healthcare practices have succeeded in reducing the risk to patients while others have not.
06	There is a gap in patient safety and existing prevention tools and strategies are not widely implemented.
07	Homogeneous reporting is absent, thus lessons learned from incidents are limited.

At the macro level, recognizing patient safety, investigating, assessing, and providing solutions for such a healthcare system was one of the WHO objectives. Therefore, an international alliance for better patient safety was started by the WHO in 2004, which was then followed by the global initiatives of 'clean care is safer care' as HH was one of the five major actions in this campaign [24]. Although many counties were committed to patient safety initiatives and have signed the formal statement pledging implementation of these strategies, in reality, and practice it is questionable, as this might need a universal auditing and a comprehensive evaluation. Every healthcare system in the world should strive to adhere to the recommendations of WHO regarding the minimum criteria for HH improvement strategy [25]. These criteria change in system, training, and education, monitoring of HH practices and performance feedback, reminders in the workplace, and having a HH and patient safety culture.

Additionally, another study was about testing the usability and reliability of the WHO HH self-assessment framework through the drafting of the tool method [26]. Drafting the HH improvement strategies of the WHO was developed by experts, and 42 facilities were asked to answer the pre-test questions about the drafts and provide feedback. Although the sample size was too small and that could affect the generalisability and transferability of the study, most of the sample participants were experts from the 'clean care is safer care' network; this would be ensuring different experiences from different countries were captured. The study of Stewardson et al. resulted in proving the usability and reliability of the WHO 'HH self-assessment framework' by all of the participants. Therefore, it could be suggested that through WHO HH guidelines, implementing a comprehensive and sustainable HH program within any healthcare system could be achieved globally and would improve healthcare workers’ HH adherence.

In 2007, an online survey was conducted by WHO using a structured questionnaire to promote HH, which was then repeated in 2009 aiming to identify factors that could be utilized during HH planning activities [27]. A survey is a way used to collect data to "describe, compare, or explain knowledge, attitudes or behavior" [28]. While the main disadvantage of the mailed questionnaires according to Fain is less of a response rate; in this survey, the return rate was 18 out of 20 and 38 out of 38 countries in 2007 and 2009, respectively, and that is considered to be excellent in surveys. However, the survey resulted in indicating some barriers to HH promotion initiatives in some countries such as weak healthcare systems, inadequate support from a healthcare facility, inadequate support by policymakers, lack of supplies, infrastructure weaknesses, lack of finance, and not being considered a priority. It could be suggested, however, that WHO HH guidelines should be applied as a basis of providing 'safer care through clean care' and should be monitored to ensure sustainability and accountability. Alternatively, benchmarking the GCC countries' HH policies and other IC policies like Oman’s HH policy from their regional perspective 'middle level' with other international viewpoints at the 'macro level' would have its benefits concerning other countries' trials to keep staff adhering to HH practice [29]. Thus, the need for research studies at the macro level administered by the WHO is of high value and should importantly be considered to form a unified and structured HH policy and/or guideline so that it can then be examined and re-examined in a different context worldwide to suit different nations.

Meso Level

At this level, the focus is on adopting a suitable HH policy within GCC countries regionally and internally or nationally in Oman. Broadly, in December 2006, the Oman Ministry of Health (MoH) and other GCC countries signed the pledge of 'Clean Care is Safer Care' to improve HH adherence [30]. Since then, the MOH has worked hard to coordinate efforts to HH adherence through the infection control department in the ministry. Arguably, the applicability of the WHO and other international HH guidelines in developing Arab and Muslim countries like Oman is doubtful because the studies have been carried out in different healthcare systems, with different beliefs and values that might make it inappropriate or difficult to transfer recommendations for healthcare workers in Oman. For example, using alcohol-based hand rub by some Muslim healthcare workers has been hesitantly rejected from the religious perspective [31]. Although alcohol ingestion is forbidden in Islam, it does not mean it is prohibited to be used for HH and health, because using "alcohol as medical agent is permitted in Islam." Therefore, utilizing the 'meso' (regional and/or national) level to undertake research studies about best practices of HH in Oman and other GCC countries with the same values is an essential and a prerequisite issue to reform and adapt suitable HH guidelines and policy for a particular area like Oman. Hence, many personal interpretations of the different religious or cultural objections would be clarified.

The main goal of the meso level in HH policy is to make macro policy (i.e. WHO HH guidelines) appropriate for healthcare workers in Oman and other GCC countries. As a result, on January 1, 2009, the MoH of the entire GCC countries adopted and edited their first infection prevention and control manual through the Executive Board of the Health Ministers’ Council. The manual contains the whole IC policies numbered according to each policy title or description [21]. The manual’s policies and process illustrate how to perform the following procedures correctly: HH technique, aseptic technique, medical waste management, employee health, and management of patients with multidrug-resistant organisms, and include other IC policies. Moreover, the whole document of HH policy in the GCC manual is congruent with the WHO guidelines and there are many commonalities with other developed countries such as the National Health System (NHS) HH policy in the UK. Through this manual, IC policy generally and HH policy particularly has attempted to unify the practices among healthcare workers in Oman as well as in other GCC countries. The GCC manual was developed to incorporate the: standards of the Joint Commission for Accreditation of Healthcare Organisation (JCAHO), Joint Commission International Accreditation (JCIA), recommendations of the CDC, and the guidelines of WHO; and many other current references that reflect the best practice.

One of the essential goals at this level is to find how to improve the HH practice in specific regions, and this could be achieved through research and evidence-based practice. In the UK, for instance, a systematic review study was undertaken by Gould et al. aiming to review the factors that improve HH adherence and its results in reducing healthcare-associated infection [32]. An electronic search time scale was used starting from 2006 up to 2009; thus different databases and search engines were used through the Cochrane Library. A systematic review is considered to be at the top of the hierarchy of evidence and used to analyze, synthesize, and evaluate a particular topic through particular research studies [33-34]. However, an inclusive search strategy, followed by appropriate evaluation, strong evidence for factors leading to HH adherence was not found in the article by Gould et al. Thus, future research studies about factors and/or interventions that improve HH adherence were suggested by Gould et al. in 2011. The significance of these types of studies at the meso level is very important because through these studies many factors that may help in better HH adherence by healthcare workers could be found. Hence, it could be suggested that GCC countries should carry out several research studies in their particular countries periodically.

Furthermore, a HH behavior measurement tool was developed through a longitudinal observational study by McAteer et al. [35]. These types of studies observe and collect data about a specific subject over a long period. This observational study took place on an intensive therapy unit over eight months and before the study, a pilot observation was performed over three months using the Geneva HH observation tool (macro-level). This offers a validity of the observational tool because pilot studies are essential to evaluate the instruments and correct any problems before implementing the actual study. However, 1191 HH observations and HH behaviors were observed and the overall adherence to HH increased significantly from 80% to 98% and the probability (p) value was less than 0.001 (p>0.001) (McAteer et al. 2008). The p-value refers to "the probability that an event will occur in a given situation," and it should not exceed 0.05 [36]. Thus, this study has proved the usability of the Geneva HH observation tool in improving the HH policy at the meso level through regional healthcare systems. Although researchers collected data about the same healthcare providers (nurses) over some time, longitudinal observation studies are "threatened by testing effects, with subjects being tested (observed) repeatedly." Therefore, further research studies using different observation tools would be beneficial in providing different perspectives in HH and may provide different behavioral approaches towards HH adherence.

Micro Level

At this level, the focus is on the practicality of the HH policy in hospital settings. The gap between HH practice and the factors that lead to healthcare providers' non-compliance with HH has been a constant theme in WHO [37]. While it has been agreed that HH policy should be applied in every healthcare setting, the accommodation or adoption of it should be proven through research studies that should be undertaken in all hospital settings (WHO 2009a). The use of water and soap, and alcohol-based hand rub in improving HH has arguably been proven through clinical trial studies but under specific influences such as WHO guidelines and other international/national HH policies; this is because most of the researchers attempted to use such a guideline or policy (i.e. WHO-HH guidelines) as basic criteria in their observation and assessment to collect data in some studies [38-39].

A recent randomized control trial (RCT) study was undertaken by Chow et al. (2012) at a tertiary hospital setting of 1300 beds, which can be considered at a micro level, to test the effectiveness of different HH protocols (from macro and/or meso levels), specifically about alcohol hand based hygiene [40]. In this study, 60 nurses and 60 doctors were randomized into three groups of 40 participants in each group, and each group tested one protocol. The participants were pre-tested and trained on the three protocols according to the CDC policy (macro or meso level). Although the study was double-blinded as the participants did not know who would test which protocol as the protocols were sealed, which offers credibility to the study and protects it from bias, still there was insufficient sample size in each group because according to Fain to ensure the effectiveness of the sample size in each group through the study statistical power, which should be around 80% of the total study sample, hence, there should have been 96 participants in each group. However, the study's results could be generalized as the results share the same conclusion of HH policy and study participants were representative of health care workers in general; this was acknowledged by the researchers in the generalisability part of the study offering honesty. However, the results of the study were statistically significant in reducing hand-associated infections because the p-value was less than 0.01 (p>0.01). Nonetheless, alcohol-based hand rub, which covers all the hand services was best and faster in reducing hands infections with a median of 26.0 seconds compared with seven steps hand rubbing (median of 38.5 seconds), and chlorhexidine hand washes protocols (median 75.5 seconds). The study concluded that "alcohol hand rubbing protocols are as efficacious as chlorhexidine. Alcohol hand rubbing covering all hand surfaces is the most time-tested protocol found effective in routine patient care activities in busy general wards". These results could be seen as evidence of the importance of testing different protocols and policies from the macro and/or meso level at the micro level within hospital settings. Therefore, the results of such studies at the micro level could be taken as a logical underpinning rationale to change or update such policies up to higher levels, such as meso or even macro level, because these studies were carried out in a clinical setting and their functionality was proven to be reasonable.

**Figure 1:**
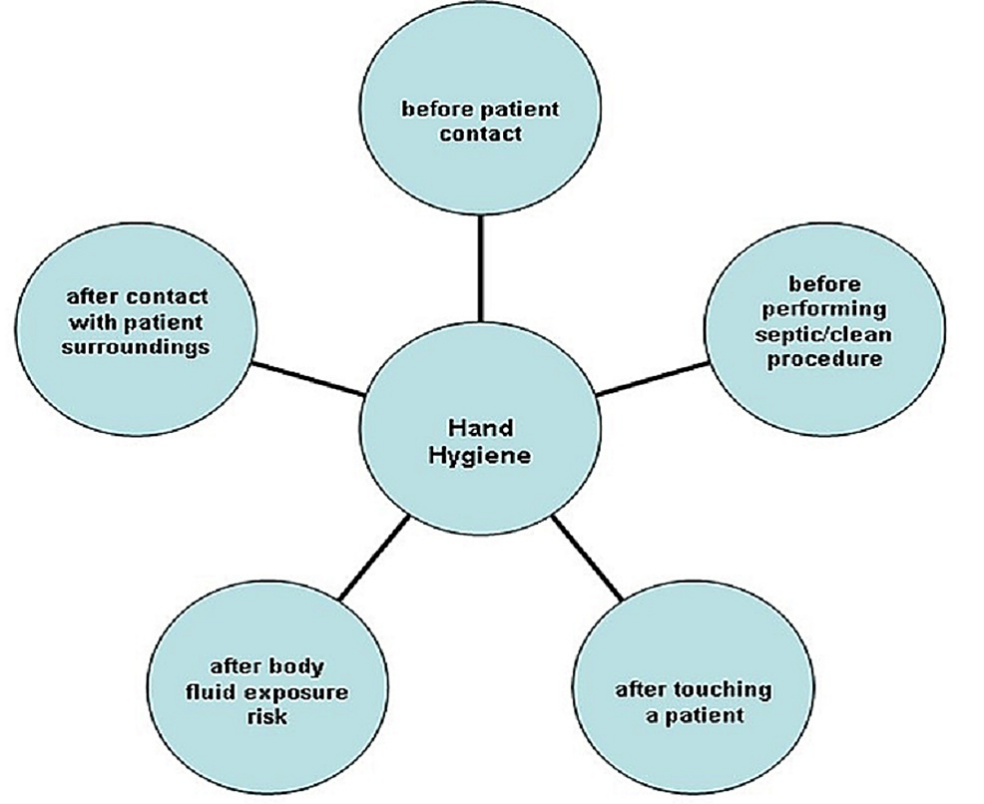
Five Moments for Hand Hygiene

Several research studies have proven low adherence to HH policy and guidelines in general healthcare settings as well as critical care settings due to different reasons such as lack of policy and guidelines, lack of knowledge, high workload, lack of training about HH, scarcity of resources, and ineffective leadership and management [41-46]. One observational study design was carried out by Alsubaie et al. in five ICUs in Saudi Arabia, aiming to estimate the adherence of HH among healthcare workers and to examine the factors associated with non-adherence [45]. Saudi Arabia is one of the GCC countries that used to share the same IC policies generally as mentioned above (meso level). However, despite the undoubted importance of such studies in the GCC countries, relatively very few studies found anything about HH among these countries. Therefore, attention should be given to improving and adopting such policies through increasing research studies in countries with the same beliefs and values rather than converting other Westernised policies to fit Arabic values without regional investigation through research. In this study, researchers utilized a macro level observation criteria when they used the WHO procedure "five moments for hand hygiene" (Figure 1) as a basis to guide the observation, performed by six trained observers. Despite the 'reactive' response that might appear if the observed participants believe they are being observed, which could affect the study results, all healthcare workers were, in fact, unaware of being observed for HH adherence, which reduced the 'reactivity' response and the staff acted as usual. However, 3940 HH chances were observed from 242 healthcare staff in the ICUs and the study resulted in 58% of the above HH observation chances being non-adherent to HH, indicating very poor adherence to HH policy and guidelines. Furthermore, this study indicated that most of the staff that adhered to HH were therapists and other technicians, and it could be argued that as those staff used to have fewer patient interactions compared with nurses and doctors, this means there were fewer handwashing chances. Above all, the study identified the main factors leading to non-adherence to HH were new staff who were not aware of HH guidelines and the high workload in some ICUs (lower nurse to patient ratio 1:2 or 1:3).

Accordingly, it could be recommended by all GCC countries to unify a HH policy training among their healthcare workers and HH policy should importantly be included in any orientation program for new healthcare personnel, and further research studies would be the keystone to gaining more knowledge about the underlying reasons for non-adherence to HH in Oman and other GCC countries.

Limited resources and the opportunity cost

Whilst scarcity of resources is a worrying issue in healthcare settings worldwide, allocating these limited funds to maximize the benefits of the patients and the society as a whole through priority setting plans remains one of the fundamental concerns of WHO and healthcare economists [47-48]. However, one of the resources that have been prioritized by WHO and followed by many countries is healthcare policy as a guideline of meeting a standardized practice according to current evidence and research studies. HH policy is considered to be one of the most important policies that reduce healthcare costs since there is a remarkable reduction in mortality and morbidity rates in the global healthcare setting. This is because spending on HH policy improvement 'as an input' by undertaking research studies to reduce healthcare-associated infections and improve the quality of care 'as an output,', is a valuable issue to reduce the cost of spending resources on treating and/or managing the consequences of non-adherence to HH.

Overall, it would be wise to understand that the precise purpose of HH and other healthcare policies is not to apply a standardized procedure, nor to reduce the cost only, but the appropriate and efficient use of the resources through need priority setting. However, the efficient use of resources concerning HH policy could be achieved using the concept of 'opportunity cost.' Opportunity cost is the cost of any action that could be considered the next best-valued alternative [19]. Undertaking research studies about HH (to reduce hospital-related infections, which would reduce mortality and morbidity rates) instead of spending resources on other research studies could be forgone (opportunity cost) as the next best alternative. Thus, moving some resources from different programs and utilizing them in developing HH policy and its related procedures would provide the greatest benefits and this should continue until no further funds or resources could be moved or until more funds are provided for the HH policy.

## Conclusions

This paper identifies the importance of examining the impact of HH policy and patient safety culture on improving healthcare workers' adherence to HH practice generally and in critical care settings specifically. This study focused on three important levels in healthcare policy: macro (international policy), meso (regional and/or national policy), and micro (in hospital settings). By adhering to HH policy, there is improved culture of safety in an organization. This is possible by continuous awareness programs demonstrating the dramatic effect of HH practice on improving patient safety and quality care. Understanding the importance of a HH policy by healthcare workers would allow them to prioritize it in their planning. The healthcare leaders can strengthen their healthcare system by improving the infrastructures, providing adequate support and resources, providing comprehensive monitoring and evaluation of patient safety initiatives. The MoH in Oman should develop a program to monitor and measure the impact of HH policy and guidelines on staff adherence to HH practice and to identify the factors that lead to poor HH practice. This could be done by deploying further resources to conduct research studies in the clinical area.
